# Body mass index and workplace bullying: exploring causality in two general population Norwegian surveys, the HUNT study and the Norwegian Mother, Father and Child Cohort Study (MoBa)

**DOI:** 10.1136/bmjph-2025-003665

**Published:** 2026-06-11

**Authors:** Amanda M Hughes, Gunnhild Åberge Vie, Alexandra Havdahl, Doriane Mignon, Christina Hansen Edwards

**Affiliations:** 1 MRC Integrative Epidemiology Unit, University of Bristol, Bristol, UK; 2 Population Health Sciences, Bristol Medical School, University of Bristol, Bristol, UK; 3 General Practice Research Unit, Department of Public Health and Nursing, Norwegian University of Science and Technology, Trondheim, Norway; 4 PsychGen Centre, Division of Public Health and Prevention, Norwegian Institute of Public Health, Oslo, Norway; 5 Research Department, Lovisenberg Diaconal Hospital, Oslo, Norway; 6 HOPE, School of Health Sciences, The University of Manchester, Manchester, UK; 7 Department of Economics, Norwegian University of Science and Technology, Trondheim, Norway; 8 Division of Mental Health Care, St Olavs Hospital Universitetssykehuset i Trondheim, Trondheim, Norway

**Keywords:** Obesity, Body Mass Index, Occupational Medicine

## Abstract

**Introduction:**

To interrogate whether reported associations between body mass index (BMI) and workplace bullying (WPB) reflect an impact of weight on bullying, an impact of bullying on weight or confounding.

**Methods:**

We use data from working-age participants in two large, general population Norwegian surveys, the Trøndelag Health Study (HUNT, N=32 826) and the Norwegian Mother, Father and Child Cohort Study (MoBa mothers, N=14 076). To interrogate causal mechanisms, we apply genetic instrumental variable analysis (Mendelian randomisation) and, in HUNT only, bidirectional longitudinal analysis.

**Results:**

In cross-sectional analysis in both surveys, participants with a higher BMI were slightly more likely to report concurrent WPB, adjusted for confounders including socioeconomic position and psychological distress (meta-analysed absolute risk difference, ARD: 0.22% per kg/m^2^, 95% CI 0.15 to 0.29, meta-analysed OR: 1.02 per kg/m^2^, 95% CI 1.01 to 1.03). Genetic analysis found strong evidence for an impact of BMI on WPB only in MoBa, but not overall (meta-analysed ARD: 0.32% per kg/m^2^, 95% CI −0.29 to 0.93). In longitudinal analyses using HUNT data, there was less evidence that earlier BMI affected WPB onset (ARD: 0.08% per kg/m^2^, 95% CI −0.10 to 0.25, with raised risk for participants with class I but not class II obesity at baseline). However, WPB in 2006–2008 was associated with higher BMI in 2017–19 (beta: 0.16 kg/m^2^ (95% CI 0.02 to 0.29)). Stratified analyses found inconsistent evidence of age, sex and socioeconomic modification, but suggested stronger associations pre-2020.

**Conclusions:**

In two large Norwegian surveys, we found inconclusive evidence from genetic models that higher BMI increased the risk of WPB. Longitudinal analysis (possible only in HUNT) found mixed evidence that higher BMI increased risk of WPB, but clearly suggested that WPB affected BMI over time, consistent with an impact of psychosocial stress on BMI. Results underscore the negative consequences of WPB for physical as well as mental health.

**WHAT IS ALREADY KNOWN ON THIS TOPIC**

- Cross-sectional studies have shown that employees with a higher body mass index (BMI) are more likely to experience workplace bullying (WPB), but the direction of causation is not known.

**WHAT THIS STUDY ADDS**

- In two large surveys from Norway, longitudinal and genetic instrumental variable analysis found inconclusive evidence that a person’s BMI influences their risk of WPB. There was clearer evidence that experiencing WPB may cause weight gain over time.

**HOW THIS STUDY MIGHT AFFECT RESEARCH, PRACTICE OR POLICY**

- Findings add to evidence that experiencing WPB can affect employees’ physical, as well as mental, health, strengthening the case for employer and regulatory measures to tackle it.

## Introduction

 Workplace bullying (WPB) can be defined as systematic mistreatment of an employee by one or more colleagues, repeatedly and over a prolonged period. This may be from a superior, peer or subordinate, but always involves a power asymmetry between the perpetrator and victim.^1^ It can take many forms, including physical or verbal aggression, spreading rumours and social isolation,^2^ or work-related acts like overloading someone with work or denying training or promotion opportunities.^3^

Nationally representative studies indicate widespread WPB, with 1 in 10 employed adults in the UK bullied at work in the past year,^4^ and more than one in four in France.^5^ An international meta-analysis found an average prevalence of 14.7% across 14 countries.^6^ This matters for public health, given strong evidence linking WPB to mental health outcomes including depression and suicide ideation.^7^ Moreover, WPB has been shown to disproportionately affect disadvantaged groups^4 8^ and may therefore exacerbate health inequalities.

In this context, it is important to understand antecedents of WPB, to identify groups at increased risk and develop more effective interventions. A large literature has demonstrated the importance of factors at the organisational level, including leadership style, organisational culture and aspects of job design, such as demands placed on workers and role ambiguity.^9^ Other research has identified personal factors which may influence risk of experiencing WPB. Women are more likely than men to report being bullied at work,^4 9^ and some studies suggest a higher risk for younger compared with older employees.^5 9^ Recent evidence suggests higher risk for mixed or minority ethnic individuals^4^ and immigrants.^8^ Poor health may increase risk of WPB, with longitudinal studies showing higher risk of WPB for people who had worse mental^10^ or overall health^11^ at baseline. Importantly, organisational factors can modify associations with individual health.^10 11^ This demonstrates the ability of employers to act on WPB and the potential for organisation-level interventions to benefit high-risk groups.

Although weight is relatively unexplored in the WPB literature, several lines of evidence suggest having a higher body mass index (BMI) may increase risk of WPB. First, there is overwhelming evidence that higher-weight individuals face stigma and discrimination,^12^ and bullying often targets socially devalued identities.^13^ Second, weight is a key risk factor for youth bullying, with a recent cross-national survey placing it ahead of ethnicity, religion or sexual orientation.^14^ Third, higher-weight employees are more likely to report perceived discrimination within their current workplace,^15^ and many experiences considered discrimination, for example, being denied a promotion, could also be interpreted as WPB.^3^

Cross-sectional studies report positive associations of BMI and WPB.^16^,^18^ However, their design limits understanding of causal mechanisms, because body weight may be a cause or consequence of WPB. Experiencing WPB is highly stressful, and psychosocial stress can lead to weight gain, through direct biological mechanisms and by influencing behaviours around eating and physical activity.^19^ Moreover, longitudinal work suggests weight-related discrimination can cause weight gain over time.^20^ Cross-sectional observational studies are often impacted by residual confounding, due to unmeasured or partially captured factors which influence weight and WPB independently (such as socioeconomic factors, health, childhood adversity or personality traits).

Bidirectional longitudinal designs, which are better able to disentangle causal mechanisms, point to reciprocal relationships between WPB and mental health^21^ and sickness absence.^22^ One longitudinal study has examined WPB in relation to weight, finding that onset of WPB predicted greater weight gain over time.^23^ In the subset of participants with necessary data, there was also suggestive evidence that increase in BMI predicted onset of WPB (OR=1.15; 95% CI 0.98 to 1.36), consistent with a bidirectional relationship. However, longitudinal designs can still be affected by residual confounding from time-varying confounders.

Using data from two large general population studies from Norway, we explore associations between BMI and bullying or harassment at work, applying multiple approaches to interrogate causal directionality. First, we apply a genetic approach known as Mendelian randomisation (MR),^24^ instrumenting BMI with an index of genetic variants associated with BMI.^25^ Under specific assumptions, MR intends to estimate causal effects. Unlike BMI itself, genetic variants are assigned at conception and cannot change in response to environmental factors or psychosocial stress. Consequently, associations of outcomes with genetic instruments, unlike with BMI itself, are robust to reverse causation and classical confounding.^24^ MR has been widely applied to investigate relationships of BMI with social^26^ and health-related outcomes.^27^ MR rests on three key assumptions: that the genetic variants used as instruments associate with the exposure (the relevance assumption); that they share no common cause with the outcome (the independence assumption); and that they do not affect the outcome except through the risk factor (the exclusion restriction assumption).^24^

Second, we conduct longitudinal analyses, using repeated measures of BMI and WPB in the same individuals over an 11-year period, to disentangle the impact of BMI on WPB, and of WPB on BMI, over time. Evidence on weight-related stigma and discrimination suggests that impact of BMI on bullying may be non-linear, with substantially increased risk above a certain threshold of body size.^28^ We therefore investigate relationships using continuous and categorised BMI, apart from in MR analyses where substantially larger sample sizes are required to investigate non-linear effects.^29^ As possible effect modifiers, we consider employee sex and age, given stronger evidence linking BMI to workplace outcomes for women and younger workers,^15 30^ employee socioeconomic position, given varying attitudes about weight across social groups,^31^ and year of data collection.

To our knowledge, this is the first application of MR to this question. It therefore adds considerably to understanding of mechanisms linking BMI and WPB.

## Methods

This study employed a mixed research design, comprising: (1) an observational, cross-sectional component in the Trøndelag Health Study (HUNT) and the Norwegian Mother, Father and Child Cohort Study (MoBa), (2) a quasi-experimental, instrumental-variable MR component in HUNT and MoBa and (3) an observational, bidirectional longitudinal component in HUNT.

### Participants

The HUNT is a longitudinal cohort study primarily of adults aged 20+ living in the former county of Nord-Trøndelag (online supplemental methods).^32 33^ At HUNT3 (2006–2008) and HUNT4 (2017–2019), all residents aged 20+ were invited to attend a clinical examination and complete questionnaires. The target sample for main cross-sectional analyses comprised all individuals who, at HUNT3 or HUNT4, were in paid employment and aged 30–66 years (N=43 102). This age range was chosen to exclude participants above Norway’s retirement age, and because those aged <30 were not asked about WPB. We excluded individuals lacking genetic data, BMI or information on occupation, WPB or psychological distress (online supplemental methods, online supplemental figure 1), leaving an analytic sample of N=32 826. For longitudinal analyses, the target sample comprised all participants of HUNT3 and HUNT4 who were employed and aged 30–66 years at both surveys (N=14 698). We excluded individuals lacking data on BMI or WPB from either survey, or occupation or psychological distress at baseline (HUNT3), leaving an analytic sample of N=9234 (online supplemental figure 2).

MoBa is a population-based pregnancy cohort study over 114 500 children, 95 200 mothers, and 75 200 fathers conducted by the Norwegian Institute of Public Health.^34^ Participants were recruited from all over Norway from 1999 to 2008, with 41% of all pregnant women invited consenting to participate (see online supplemental methods). When the study children were aged 14 years, mothers completed a questionnaire which asked about WPB, height and weight. The MoBa target sample comprised all unique mothers who, at completion of this questionnaire, were of working age and employed (N=16 947). We excluded mothers without quality-controlled genotype data (online supplemental methods)^35^ or information on BMI, WPB, psychological distress, age or education, leaving an analytic sample of N=14 076 (online supplemental figure 3).

### Measures

#### Phenotypic information in HUNT

As part of a wider questionnaire, participants aged 30–69 of HUNT3 (in 2006–2008) and HUNT4 (in 2017–2019) were asked ‘Are you bullied/harassed at work?’ and could respond: ‘No, could say never’, ‘No, seldom’, ‘Yes, sometimes’ or ‘Yes, often’. To allow comparison with bullying in MoBa, this was dichotomised as no or almost no bullying (‘No, could say never’) and bullying (all other categories). Height and weight were measured at a clinical examination.^32^ Sex recorded at birth and age at survey participation were derived from registry data. Given the focus on the workplace environment, we included as a socioeconomic indicator a 10-group classification of current occupational role and sector (categories shown in table 1), based on the International Standard Classification of Occupations-88.^36^ To capture psychological distress, we included the 14-item Hospital Anxiety and Depression Scale (HADS)^37^ (online supplemental methods).

**Table 1:** Descriptive characteristics of HUNT cross-sectional analytic sample (N=32 826)

	Mean	SD	Range
Age in years	49.4	9.7	30–66
BMI (kg/m^2^)	27.2	4.4	12.1–59.5
HADS score	7.2	5.4	0–42
			%
Sex	Female		54.9
	Male		45.1
Occupation	Legislators, senior officials and managers		8.6
	Professionals		13.2
	Technicians and associate professionals		25.2
	Clerks		6.0
	Service workers, shop and market sales workers		21.1
	Skilled agricultural and fishery workers		5.7
	Craft and related trade workers		9.4
	Plant and machine operators and assemblers		6.5
	Elementary occupations		3.8
	Armed forces, unspecified occupations		0.5
Workplace bullying	No, could say never		85.6
	No, rarely		11.9
	Yes, sometimes		4.1
	Yes, often		0.4
BMI, categorised	Recommended weight (18.5–24.9 kg/m^2^)		31.9
	Overweight (25.0–29.9 kg/m^2^)		44.7
	Obesity I (30.0–34.9 kg/m^2^)		17.5
	Obesity II (≥35.0 kg/m^2^)		5.5
	Underweight (<18.5 kg/m^2^)		0.4
Survey	HUNT3 (2006–2008)		53.3
	HUNT4 (2017–2019)		46.8

All individuals who were of working age (<67), in paid employment, with available genetic data, and complete data on BMI, workplace bullying and covariates at either HUNT3 or HUNT4.

BMI, body mass index; HADS, Hospital Anxiety and Depression Scale; HUNT, the Trøndelag Health Study.

#### Phenotypic information in MoBa

As part of a questionnaire completed when study children were aged 14, MoBa mothers were asked ‘Have you been bullied/harassed at your workplace?’ and could respond: ‘very rarely or never’, ‘rarely’, ‘sometimes’, ‘fairly often’, ‘very often or always’ or ‘not relevant’. Since mothers who were not currently employed had been excluded, those who answered ‘not relevant’ may work in roles where there is little contact with others. This small group (N=344, around 2.4% of the sample) were excluded from analyses (online supplemental figure 2). To allow comparison with bullying in HUNT, this was dichotomised as no or almost no bullying (‘Very rarely or never’) and bullying (all other categories). This questionnaire was completed between 2016 and 2022, depending on the birth year of the child. Height and weight were self-reported within the same questionnaire; values >4 SD from the mean were treated as errors and removed. Mother’s age was derived from recorded age at the study child’s birth in the Medical Birth Registry of Norway. Information on mothers’ occupation was not available, so as a socioeconomic indicator, we used the mother’s highest level of formal education from administrative data (online supplemental methods). To capture psychological distress, we used a scale based on 14 selected items from the Hopkins Symptoms Checklist-25 (online supplemental methods).

#### Genetic information

Polygenic scores (PGSs) were derived using data on single nucleotide polymorphisms (SNPs) associated with BMI in a genome-wide analysis (GWAS) of 700 000 adults of European ancestry.^25^ PGSs were derived by summing each participant’s number of BMI-increasing alleles, multiplied by the corresponding effect size from the GWAS (online supplemental methods). Details of genetic quality control and imputation in HUNT^38^ and MoBa^35^ are available elsewhere. To account for ancestral clustering, we included the first 20 principal components (PCs) of ancestry and genotyping batch in genetic models. For consistency, these were also included in cross-sectional non-genetic models.

### Statistical analysis

Linear probability models (LPMs) were used to explore cross-sectional associations (HUNT: N=32 826, MoBa: N=14 076) between BMI and reported WPB. LPMs are commonly used in fields including economics because they permit direct comparison between ordinary least squares (OLS) regression and instrumental variable regression results. In contrast, instrumental-variable logistic regression cannot be straightforwardly applied using a two-stage regression method without overestimating precision.^39^ Meanwhile, absolute risk differences (ARDs) are more intuitive to interpret than results of probit models. LPMs estimate the absolute difference in probability of an outcome (ARD) per unit change in an exposure, usually expressed as a percentage. Compared with ORs, they have the advantage of being directly comparable across models (eg, between non-genetic and MR models) and subgroups (eg, age bands). Where possible (ie, for non-genetic models) we also present results from logistic regression on the OR scale. In HUNT and MoBa, an initial non-genetic model explored the cross-sectional association between BMI and WPB, adjusted for participant age, sex (in HUNT), ancestry PCs and genotyping batch. A second model additionally adjusted for psychological distress (HADS in HUNT, Hopkins Checklist in MoBa) and a socioeconomic indicator (occupation in HUNT, educational qualifications in MoBa). Third, Stata’s ivregress was used to run a linear probability, two-stage least squares MR model in which BMI was instrumented by the BMI PGS, adjusted for age in years, sex (in HUNT), ancestry PCs and genotyping batch. For comparability, we performed complete-case analysis using the same exclusion criteria for all cross-sectional BMI models (online supplemental figures 1 and 3). Finally, a random-effects meta-analysis was performed of cross-sectional results from the two studies using Stata’s metan package.

Longitudinal analyses in HUNT (N=9234) explored associations between (1) the ‘forward direction’: BMI at HUNT3 (in 2006–2008) and WPB at HUNT4 (in 2017–2019) using LPMs and (2) the ‘reverse direction’: WPB at HUNT3 (in 2006–2008) and BMI at HUNT4 (in 2017–2019), using linear regression. For comparability, we performed complete-case analysis in the same analytic sample for all longitudinal models in both directions (online supplemental figure 2). For both the forward and reverse direction, an initial model adjusted for age in years and sex. A second model added occupation and psychological distress at baseline, and a third model added the outcome (WPB, or BMI) measured at baseline. For the subset of participants with BMI measurements from HUNT2 (1995–1997 (N=7031)), we also ran a forward-direction model which included HUNT2 BMI as a covariate. This explored the impact of BMI increase between HUNT2 and HUNT3 on onset of WPB between HUNT3 and HUNT4.

Because longitudinal analyses were restricted to people employed at HUNT3 and HUNT4, results would not capture experiences of people who left the labour market between the two surveys, including in response to WPB. We therefore examined whether participants who experienced WPB at HUNT3 (in 2006–2008) were more likely to leave the labour market by HUNT4 (2017–2019). For this, we ran an LPM adjusted for baseline age, sex, occupation, psychological distress and BMI, using all participants employed at HUNT3 (in 2006–2008), of working age at both timepoints, and with data on WPB, employment status and baseline covariates (N=13 296).

To explore nonlinear effects with BMI, we ran additional non-genetic models with BMI categories (<18.5 kg/m^2^, 18.5–24.9 kg/m^2^, 25.0–30.0 kg/m^2^, 30–34.9 kg/m^2^, ≥35 kg/m^2^). It was not statistically feasible to run non-linear MR models, which require substantially larger sample sizes.^29^ We ran additional MR models using two-sample methodology (Inverse Variance Weighted, MR-Egger, MR-mode and MR-median) which rely on different assumptions and can be used to explore robustness of MR results.^40^ Stratified models examined associations by age (30–44 vs 45+) and sex (female vs male, HUNT only). To explore differences by year of data collection, HUNT participants were stratified by survey (2006–2008 vs 2017–2019), and MoBa participants by year of questionnaire completion, splitting at the median (2016–2019 vs 2020–2022). To explore socioeconomic differences, HUNT participants were stratified by occupational skill level (lower vs higher) and MoBa participants by whether they attended higher education (online supplemental methods). Analysis was performed using Stata V.19 (HUNT analyses) or 17 (MoBa analyses).

### Public and patient involvement

This work formed part of a larger project on causes and consequences of weight-related stigma which ran from April 2023 to April 2025. Prior to the start of the project, an advisory group of 7 individuals with lived experience of obesity were recruited via Obesity Voices. This could only occur once we had secured funding, so broad aspects of the work (eg, the secondary data sources used in the project) had been decided. Through a series of online focus group sessions, the advisory group were involved in refining research questions to reflect the group’s perspectives and priorities and, later, in interpretation of results. For this analysis, a notable change was including sex-stratified analyses throughout and adding a second, all-female study population to adequately separate workplace experiences of men and women.

## Results

14.4% of HUNT participants, and 9.4% of MoBa participants, reported having been bullied at work in the past year (tables1  2). Within HUNT, male and female participants were equally likely to report bullying (14.3% vs 14.4%). In HUNT and MoBa respectively, participants’ mean BMI was 27.2 kg/m^2^ and 25.2 kg/m^2^, and partial R^2^ values showed that the PGS explained 3.6% of variation in BMI in both surveys (first-stage F-statistics: 1224 and 526). Within the HUNT longitudinal sample, WPB was reported by 15.4% of participants at HUNT3, and 14.6% of participants at HUNT4 (online supplemental table 1).

**Table 2:** Descriptive characteristics of MoBa cross-sectional analytic sample (N=14 076)

	Mean	SD	Range
Age in years	44.7	4.3	31–60
BMI (kg/m^2^)	25.2	4.3	13.9–51.8
Hopkins checklist score	5.0	5.6	0–42
Year questionnaire completed*	2019	1.6	2016–2022
			%
Sex	Female		100
	Male		0
Highest education qualification	Primary		0
	Lower secondary		1.9
	Upper secondary (basic)		1.1
	Upper secondary (final year)		15.1
	Post-secondary not higher education		2.1
	Higher education (undergraduate)		56.3
	Higher education (graduate level)		21.2
	Higher education (postgraduate)		2.1
Workplace bullying	Very rarely or never		90.6
	Rarely		4.9
	Sometimes		2.2
	Fairly often		0.5
	Very often or always		1.8
BMI, categorised	Recommended weight (18.5–24.9 kg/m^2^)		55.5
	Overweight (25.0–29.9 kg/m^2^)		30.2
	Obesity I (30.0–34.9 kg/m^2^)		10.1
	Obesity II (≥35.0 kg/m^2^)		3.3
	Underweight (<18.5 kg/m^2^)		0.9

All mothers who, at the time of completing the questionnaire, were of working age (<67) and in paid employment, with available genetic data and complete information on BMI, workplace bullying and covariates.

* Estimated as 14 years after the study child’s year of birth.

BMI, body mass index; MoBa, Norwegian Mother, Father and Child Cohort Study.

### Cross-sectional association between BMI and WPB

In both HUNT and MoBa, in a phenotypic model adjusted for sex, age, and genetic covariates, there was a small but statistically significant association between BMI and WPB (table 3), which attenuated slightly with adjustment for occupation and psychological distress (meta-analysed ARD: 0.22%, 95% CI 0.15% to 0.29%), meta-analysed OR: 1.02, 95% CI 1.01 to 1.03). Estimates from MR models were less precise. MR models suggested a causal impact of BMI on WPB only in MoBa (ARD: 0.65%, 95% CI 0.06% to 1.25%) but not in HUNT (ARD: 0.03%, 95% CI −0.45 to 0.51) and not when MR results were meta-analysed (ARD: 0.32%, 95% CI −0.29% to 0.93%). Results of alternative MR models using two-sample methodology (online supplemental table 2) were consistent with main MR models but estimates less precise. In non-genetic models using categorised BMI, significantly raised risk and odds of WPB increased steadily through higher BMI categories in both surveys; there was no strong evidence of a nonlinear association between BMI and WPB, although estimates for the underweight group were imprecise (table 4).

**Table 3:** Cross-sectional associations models between BMI categories and workplace bullying, HUNT and MoBa

HUNT (N=32 826)	Model 1*	Model 1*	Model 2†	Model 2†	Model 3: MR‡
	Absolute risk difference (%)	OR	Absolute risk difference (%)	OR	Absolute risk difference (%)
BMI per kg/m^2^	0.27 (0.18 to 0.37)	1.02 (1.01 to 1.03)	0.22 (0.13 to 0.31)	1.02 (1.01 to 1.03)	0.03 (–0.45 to 0.51)
BMI category§	Absolute risk difference (%)	OR	Absolute risk difference (%)	OR	
Overweight (25.0–29.9 kg/m^2^)	1.23 (0.28 to 2.18)	1.10 (1.02 to 1.18)	1.11 (0.19 to 2.04)	1.09 (1.02 to 1.18)	
Obesity I (30.0–34.9 kg/m^2^)	2.94 (1.73 to 4.14)	1.24 (1.14 to 1.35)	2.28 (1.09 to 3.46)	1.19 (1.09 to 1.30)	
Obesity II (≥35.0 kg/m^2^)	4.14 (2.30 to 5.99)	1.34 (1.18 to 1.53)	3.21 (1.39 to 5.02)	1.27 (1.11 to 1.45)	
Underweight (<18.5 kg/m^2^)	4.21 (–1.97 to 10.38)	1.35 (0.88 to 2.06)	1.57 (–4.48 to 7.61)	1.13 (0.73 to 1.74)	

MoBa (N=14 076)	Model 1*	Model 1*	Model 2†	Model 2†	Model 3: MR‡
	Absolute risk difference (%)	OR	Absolute risk difference (%)	OR	Absolute risk difference (%)
BMI per kg/m^2^	0.27 (0.16 to 0.39)	1.03 (1.02 to 1.04)	0.22 (0.11 to 0.33)	1.03 (1.01 to 1.04)	0.65 (0.06 to 1.25)
BMI category§	Absolute risk difference (%)	OR	Absolute risk difference (%)	OR	
Overweight (25.0–29.9 kg/m^2^)	1.47 (0.38 to 2.56)	1.19 (1.05 to 1.36)	1.21 (0.12 to 2.29)	1.17 (1.02 to 1.33)	
Obesity I (30.0–34.9 kg/m^2^)	3.06 (1.41 to 4.71)	1.41 (1.17 to 1.69)	2.34 (0.70 to 3.99)	1.31 (1.09 to 1.58)	
Obesity II (≥35.0 kg/m^2^)	4.83 (2.08 to 7.58)	1.66 (1.25 to 2.21)	3.88 (1.11 to 6.61)	1.52 (1.14 to 2.03)	
Underweight (<18.5 kg/m^2^)	−0.66 (–5.81 to 4.48)	0.92 (0.48 to 1.76)	−0.88 (–5.92 to 4.22)	0.90 (0.47 to 1.75)	

Meta-analysed¶	Model 1*	Model 1*	Model 2†	Model 2†	Model 3: genetic IV‡
	Absolute risk difference (%)	OR	Absolute risk difference (%)	OR	Absolute risk difference (%)
BMI per kg/m^2^	0.27 (0.20 to 0.34)	1.02 (1.01 to 1.03)	0.22 (0.15 to 0.29)	1.02 (1.01 to 1.03)	0.32 (–0.29 to 0.93)
BMI category§	Absolute risk difference (%)	OR	Absolute risk difference (%)	OR	
Overweight (25.0–29.9 kg/m^2^)	1.33 (0.62 to 2.05)	1.12 (1.05 to 1.21)	1.15 (0.45 to 1.86)	1.11 (1.04 to 1.18)	
Obesity I (30.0–34.9 kg/m^2^)	2.98 (2.01 to 3.96)	1.29 (1.15 to 1.44)	2.27 (1.27 to 3.27)	1.21 (1.12 to 1.32)	
Obesity II (≥35.0 kg/m^2^)	4.36 (2.82 to 5.89)	1.44 (1.19 to 1.75)	3.41 (1.89 to 4.92)	1.33 (1.14 to 1.55)	
Underweight (<18.5 kg/m^2^)	1.46 (–3.27 to 6.20)	1.20 (0.84 to 1.72)	0.14 (–3.76 to 4.03)	1.05 (0.73 to 1.52)	

* Adjusted for age (years), sex (HUNT only), principal components of ancestry, genotyping batch and survey year (HUNT) or year of questionnaire completion (MoBa).

† Model 1+ psychological distress (HADS in HUNT, Hopkins Checklist in MoBa) and socioeconomic indicator (occupation in HUNT, education in MoBa).

‡ Mendelian randomisation, adjusted for age, sex (HUNT only), principal components of ancestry, genotyping batch and survey year (HUNT) or questionnaire completion year (MoBa).

§ Reference: recommended weight (18.5–24.9 kg/m2).

¶ From random-effects meta-analysis.

BMI, body mass index; HADS, Hospital Anxiety and Depression Scale; HUNT, the Trøndelag Health Study; MoBa, Norwegian Mother, Father and Child Cohort Study; MR, Mendelian randomisation.

**Table 4:** Longitudinal associations between BMI and workplace bullying, HUNT only (N=9234)

Forward: association of BMI in 2006–2008 (per kg/m^2^) and workplace bullying in 2017–2019						
	Model 1*	Model 1*	Model 2†	Model 2†	Model 3‡	Model 3‡
	Absolute risk difference (%)	OR	Absolute risk difference (%)	OR	Absolute risk difference (%)	OR
BMI per kg/m^2^	0.12 (–0.06 to 0.30)	1.01 (1.00 to 1.02)	0.08 (–0.10 to 0.25)	1.01 (0.99 to 1.02)	0.01 (–0.16 to 0.18)	1.00 (0.99 to 1.02)
BMI category (Reference: recommended weight (18.5–24.9 kg/m^2^))						
Overweight (25.0–29.9 kg/m^2^)	0.76 (–0.89 to 2.42)	1.06 (0.93 to 1.22)	0.52 (–1.13 to 2.16)	1.04 (0.91 to 1.20)	0.29 (–1.30 to 1.87)	1.03 (0.89 to 1.18)
Obesity I (30.0–34.9 kg/m^2^)	3.96 (1.70 to 6.23)	1.35 (1.14 to 1.61)	3.49 (1.23 to 5.74)	1.31 (1.10 to 1.56)	2.69 (0.52 to 4.86)	1.25 (1.04 to 1.50)
Obesity II (≥35.0 kg/m^2^)	−0.49 (–4.29 to 3.31)	0.96 (0.70 to 1.32)	−1.24 (–5.01 to 2.54)	0.90 (0.66 to 1.24)	−2.12 (–5.74 to 1.51)	0.84 (0.60 to 1.17)
Underweight (<18.5 kg/m^2^)	4.68 (–6.75 to 16.11)	1.41 (0.61 to 3.22)	1.03 (–10.32 to 12.39)	1.03 (0.44 to 2.43)	3.38 (–7.54 to 14.30)	1.29 (0.54 to 3.11)

Reverse: association of workplace bullying in 2006–2008 and BMI (per kg/m^2^) in 2017–2019			
	Model 1*	Model 2†	Model 3‡
	Difference in BMI (kg/m^2^)	Difference in BMI (kg/m^2^)	Difference in BMI (kg/m^2^)
Workplace bullying	0.55 (0.30 to 0.80)	0.46 (0.20 to 0.71)	0.16 (0.02 to 0.29)

* Adjusted for age in years, sex (HUNT only), principal components of ancestry, genotyping batch and survey year (HUNT) or year of questionnaire completion (MoBa).

† Model 1+ psychological distress (HADS in HUNT, Hopkins Checklist in MoBa) and socioeconomic indicator (occupation in HUNT, education in MoBa).

‡ Model 2+ baseline value of the outcome (workplace bullying or BMI).

BMI, body mass index; HADS, Hospital Anxiety and Depression Scale; HUNT, the Trøndelag Health Study; MoBa, Norwegian Mother, Father and Child Cohort Study.

Stratified analyses (online supplemental table 3) showed that associations in phenotypic models between BMI and WPB in HUNT were driven by male participants. Adjusted for age and PCs, likelihood of WPB was 0.59% higher per kg/m^2^ (95% CI 0.44 to 0.75) for males, vs 0.11% higher per kg/m ^2^ (95% CI −0.00 to 0.22) for females. In the MR model, estimates were less precise, and CIs overlapped (ARD, males: 0.50%, 95% CI −0.34% to 1.34%, ARD, females: −0.27%, 95% CI −0.85% to 0.31%). There was little evidence that in HUNT age (30–44 vs 45–66), occupation (lower vs higher-skilled) or year (2006–2008 vs 2017–2019) modified associations. In MoBa, stratified analyses indicated the MR estimate was driven by mothers aged 30–44 (ARD: 1.18% per kg/m^2^, 95% CI 0.36 to 2.00) rather than those aged 45–60 (ARD: 0.03%, 95% CI −0.83% to 0.89%), mothers who had not attended university (ARD: 1.98%, 95% CI 0.62% to 3.33%) rather than those who had (ARD: 0.29%, 95% CI −0.38% to 0.96%) and observations from 2016 to 19 (ARD: 1.11%, 95% CI 0.25% to 1.97%) rather than 2020–2022 (ARD: 0.13%, 95% CI −0.68% to 0.93%).

### Longitudinal association between BMI and WPB

In longitudinal analyses of female and male HUNT participants, there was little evidence that continuous BMI (in kg/m^2^) at HUNT3 (in 2006–2008) predicted WPB at HUNT4 (in 2017–2019) (table 4). In models using BMI categories, participants who at HUNT3 had a BMI corresponding to class I obesity (30–34.9 kg/m^2^) were more likely than those with a recommended BMI (18.5–25.9 kg/m^2^) to report WPB; this was robust to full adjustment (ARD=2.69%, 95% CI 0.52% to 4.86%, OR=1.25, 95% CI 1.04 to 1.50). However, there was no such increased risk for participants whose BMI at HUNT3 corresponded to overweight (25.0–29.9 kg/m^2^) or class II obesity (≥35 kg/m^2^) (table 4). Point estimates were consistent with greater risk of WPB for those in the underweight category, but CIs were wide (ARD=3.38%, 95% CI −7.54% to 14.30%, OR=1.29, 95% CI 0.54 to 3.11). Results were similar when also accounting for BMI at HUNT2 in the subset of participants with this information (online supplemental table 4).

In contrast, participants who reported WPB in 2006–2008 had a 0.55 kg/m^2^ higher BMI (95% CI 0.30 to 0.80) in 2017–2019 compared with those who did not. This attenuated, but did not disappear, with adjustment for occupation, psychological distress, and BMI at baseline (0.16 kg/m^2^, 95% CI 0.20 to 0.71). In longitudinal models stratified by baseline age, associations in both directions were qualitatively similar between strata, with CIs substantially overlapping (online supplemental table 5). In sex-stratified longitudinal analyses, point estimates suggested a stronger association between baseline BMI and WPB at follow-up for male participants, but were imprecise. After full adjustment, including for WPB at baseline, the ARD was 0.19% per kg/m^2^ (95% CI −0.11% to 0.50%) for males and −0.06% per kg/m^2^ (95% CI −0.27% to 0.15%) for females.

To explore the impact of HUNT participants leaving the labour market between HUNT3 and HUNT4, we examined whether, among participants employed at HUNT3 and of working age at both surveys, WPB predicted employment status at HUNT4. There was no evidence of an impact of WPB on employment (ARD=0.08%, p=0.92).

## Discussion

In two large, general population surveys from Norway, we found positive cross-sectional associations between BMI and risk of experiencing WPB, with probability of WPB increasing across BMI categories. This was robust to adjustment for covariates including psychological distress and socioeconomic position. In contrast, there was less evidence from longitudinal analysis, where only class I obesity (BMI 30–34.9 kg/m^2^) but neither overweight (BMI 18.5–29.9 kg/m^2^) nor class II obesity (BMI ≥35 kg/m^2^) at baseline predicted onset of bullying at follow-up. Similarly, there was inconsistent evidence from MR of an impact of BMI on WPB, with strong evidence only in one of our two surveys and not in meta-analysed results. However, longitudinal analyses did find evidence of an impact of WPB on weight gain over time. Taken together, our results suggest previously reported cross-sectional associations between BMI and WPB may in part reflect an impact of WPB on BMI, not just of BMI on WPB. However, MR models suggested an impact of BMI on WPB among MoBa mothers, and genetic estimates were imprecise, meaning small effects in other groups cannot be excluded.

In HUNT, most associations between BMI and WPB were stronger for male than female participants. This contrasts with previous evidence of stronger associations of BMI with adverse work outcomes for women.^15 30^ However, it aligns with experiences shared by our lived experience advisory group, where males described overt weight-related WPB, while females described more subtle experiences which might not always be interpreted as bullying. On the other hand, the MR estimate suggested a causal effect only among MoBa mothers. Similarly, only in MoBa was there evidence of a stronger impact of BMI on WPB for younger employees and of less socioeconomically advantaged mothers (those who did not attend university). There was no evidence of modification by age or occupation in HUNT. Genetic work to disentangle causal directionality in larger surveys, male-specific surveys, and surveys of different age groups will be required to better understand how demographic factors influence associations. In HUNT, results for 2006–2008 and 2017–2019 were very similar, but in MoBa, MR results suggested an impact of BMI on WPB only in 2016–2019 and not 2020–2022. An increase in remote working following the COVID-19 pandemic^41^ may have reduced the impact of BMI on WPB, but analysis with more recent data is required to confirm this.

This study has considerable strengths. To our knowledge, this is the first study to apply MR to investigate BMI as a risk factor for WPB. This allowed us to interrogate causality to an extent not previously possible. All analyses had large sample sizes (from N=9234 to N=32 236), and participants were drawn from two general population surveys, increasing the robustness of generalisability of findings.

A limitation is that, although HUNT is considered broadly representative of the Norwegian population on key variables such as BMI,^33^ loss to follow-up between HUNT3 and HUNT4 may have impacted representativeness in longitudinal analyses. Loss to follow-up may have impacted representativeness in MoBa, given that around 28% of mothers initially in the study completed the age 14 questionnaire. In addition, missing responses to individual questions, particularly for WPB and psychological distress, may have influenced associations. Bullying was in both surveys a relatively rare event, limiting our ability to detect small differences in risk, and there were slight differences between HUNT and MoBa in the phrasing of the bullying item. There are inherent challenges in measuring WPB, and estimated prevalence has been shown to vary between measurement approaches.^6^ In HUNT and MoBa, participants were asked explicitly about being ‘bullied’, rather than about acts commonly regarded as bullying. However, something may be considered bullying or not depending on a person’s subjective experience, including the psychological distress caused and perceived intentionality. Moreover, some people may not admit to being bullied due to associated stigma. Consequently, results may have differed if WPB had been differently assessed. Due to different data collected by the surveys, we used different psychological distress measures and socioeconomic indicators as covariates, although associations in adjusted phenotypic models were very similar across the surveys. Estimates from non-genetic models may have been impacted by residual confounding by factors other than those adjusted for, including other lifestyle factors and physical health conditions which were not captured in these datasets. BMI was based on objective measurements in HUNT, but self-reported height and weight in MoBa, and measurement error may have influenced MoBa results. There are limitations to both LPMs and logistic regression, but consistency of phenotypic results across these models strengthened robustness of conclusions. Underweight individuals may have raised risk of WPB, and our phenotypic results are consistent with nonlinear associations. However, MR does not permit investigation of nonlinear effects without much larger sample sizes, so this could not be investigated; MR estimates could therefore underestimate impact of both low and high BMI on WPB. PGS are imperfect and do not capture full genetic propensity for a trait. At the same time, MR analyses using PGS can be biased if genetic variants in the PGS influence the outcome via pathways independent of the exposure (i.e., the exclusion restriction is violated). This could happen if, for instance, BMI-associated SNPs influence perception or reporting of WPB via psychological mechanisms. It is not possible to rule out such effects, which could have inflated our MR estimates. Additionally, in genetic analyses of unrelated individuals, estimated causal effects can be affected by family-level biases which cannot be accounted for by adjustment for ancestry PCs.^27^ These processes (which violate the independence assumption) could also have impacted our MR estimates. Future work in larger family-based studies will be required to better understand these processes.

Cross-national surveys suggest a lower prevalence of WPB in Scandinavian countries than the rest of Europe,^6^ and findings may not generalise to societies where WPB is more prevalent. Analysis was restricted to people of European ancestry due to small numbers in other groups; consequently, results cannot speak to the experiences of ethnic minority groups, who also experience weight-related stigma.^42^ Finally, participants reported WPB between 2006 and 2022, and mostly between 2006 and 2019. Since the COVID-19 pandemic, the nature of work has changed profoundly, with many more people working from home. While WPB can certainly occur in this context,^41^ understanding whether risk factors for WPB have also changed will require analysis in more recent datasets. Regardless of which individual-level risk factors can increase a person’s risk of WPB, the ability and responsibility to tackle WPB lies with employers^10 11^ and policymakers who set the wider regulatory framework. Currently, only in a few European countries is bullying or harassment unrelated to protected characteristics defined, expressly prohibited and regulated in employment law. The International Labour Organization’s Convention No.190, adopted in 2019, set the first global labour standards explicitly addressing violence and harassment at work, defined as “…unacceptable behaviours and practices, or threats thereof, whether a single occurrence or repeated, that aim at, result in, or are likely to result in physical, psychological, sexual or economic harm”. To date, only 55 countries worldwide have signed it.^43^

## Conclusions

In two large, general population surveys from Norway, genetic and longitudinal analyses found inconclusive evidence that a person’s BMI impacts their risk of experiencing WPB. There was clearer evidence that experiencing WPB can increase BMI. Results add to the evidence that WPB negatively impacts physical as well as psychological health, strengthening the case for employer and regulatory measures to tackle WPB.

## Supplementary material

10.1136/bmjph-2025-003665 online supplemental file 1

## Data Availability

Data may be obtained from a third party and are not publicly available.
