# Supplementary material for: Body mass index and workplace bullying: exploring causality in two general population Norwegian surveys, the HUNT study and the Norwegian Mother, Father and Child Cohort Study (MoBa)
Source: BMJ Public Health. 2026 Jun 11;4(2):e003665. doi: 10.1136/bmjph-2025-003665 (PMC13264922; doi:10.1136/bmjph-2025-003665)
Supplement: online supplemental file 1 [file bmjph-4-2-s001.pdf]

Supplementary information

Supplementary Methods

1. Details of the HUNT study
2. Details of the MoBa study
3. Note on sample overlap between HUNT and MoBa
4. Details of inclusion and exclusion of participants
5. Details of phenotypic variables used in this analysis
6. Details of polygenic scores and two-sample MR analyses

Supplementary Figures

1. Exclusions from the HUNT analytic sample (cross-sectional analyses)
2. Exclusions from the MoBa analytic sample (cross-sectional analyses)
3. Exclusions from the HUNT analytic sample (longitudinal analyses)

Supplementary Tables

1. Descriptive characteristics of HUNT longitudinal sample
2. Results from two-sample MR analyses
3. Cross-sectional analyses, stratified
4. Longitudinal associations between BMI at HUNT3 and workplace bullying at HUNT4, also adjusting for BMI at HUNT2
5. Longitudinal analyses, stratified

Supplementary Methods

1. Details of the HUNT study

The Trøndelag Health Study (the HUNT Study) is a population-based cohort study of the adult population in Trøndelag County, Norway. So far, four surveys have been conducted; HUNT1 in 1984-86, HUNT2 in 1995-97, HUNT3 in 2006-08 and HUNT4 in 2017-2019(1). The entire adult population in the former Norwegian county Nord-Trøndelag was invited to participate in each of the four surveys. In 2019, the study expanded to cover both regions of Trøndelag County, Nord-Trøndelag and Sør-Trøndelag, although residents of Sør-Trøndelag only completed questionnaire information, so their data could not be used in this analysis. All participants gave written informed consent for participation and the use of data in research. The latter three study waves have included blood samples, and genotyping data are available for a total of approximately 88 000 participants. Additionally, adolescents aged 13-19 have been invited to participate in surveys from HUNT2, and in HUNT4. Further details on measurements included at each survey are available elsewhere(1,2). Research data are available upon approval by The Regional Committees for Medical and Health Research Ethics and HUNT Research Center. Data can also be linked to other public or health registries using an individual identification number, subject to additional approval by the data owners. The genotyping in HUNT was approved by the Regional Committee for Ethics in Medical Research, Central Norway (2014/144 and 2018/1622). People of non-European ancestry were excluded from the genetic dataset because small numbers precluded meaningful analysis. Data and samples are stored by HUNT Databank according to the Norwegian National Regulations on Population-Based Health Surveys. The current project was approved by the Regional Committee for Ethics in Medical Research, Central Norway (2016/552).

2. Details of the MoBa study

The Norwegian Mother, Father and Child Cohort Study (MoBa) is a population-based pregnancy cohort study conducted by the Norwegian Institute of Public Health(3). Participants were recruited from all over Norway from 1999 to 2008. The women consented to participation in 41% of the pregnancies. The cohort includes approximately 114,500 children, 95,200 mothers and 75,200 fathers. All the mothers and fathers provided written informed consent at recruitment during the pregnancy. Blood samples were obtained from both parents during pregnancy and from mothers and children (umbilical cord) at birth(4). Genetic data was quality controlled using the MoBaPsychGen pipeline for European ancestry genotype data(5). The first child was born in October 1999 and the last in July 2009. The current study is based on version 12 of the quality-assured data files released for research in January 2019. The establishment of MoBa and initial data collection was based on a license from the Norwegian Data Protection Agency and approval from The Regional Committees for Medical and Health Research Ethics. The MoBa cohort is currently regulated by the Norwegian Health Registry Act. The current study was approved by The Regional Committees for Medical and Health Research Ethics (2016/1702). The Medical Birth Registry (MBRN) is a national health registry containing information about all births in Norway. For this analysis, if a mother had multiple children in the study, we used information reported when the eldest child was 14.

3. Note on sample overlap between HUNT and MoBa

A small number of people who met eligibility criteria for both HUNT and MoBa may have participated in both surveys. The exact number is not known but overlap in our study is likely to

be minimal: municipality of residence records indicate only 3.3% of our MoBa sample would have been eligible for HUNT3 or HUNT4.

4. Details of inclusion and exclusion of participants

HUNT. For cross sectional analyses, we excluded people lacking genetic data, measured BMI or reported occupation (from the clinical examination), or self-reported information about WPB or psychological distress. The largest exclusion was for missing data on workplace bulling or psychological distress, because at both HUNT3 and HUNT4 only three-quarters of participants who attended the clinical examination completed the relevant questionnaire (Supplementary Figure 1). For individuals who met all inclusion criteria at both surveys (N=8,959), information was taken from HUNT3 or HUNT4 according to whether the participant ID was odd or even. For longitudinal analyses, again, most exclusions were for missing data on WPB and psychological distress (Supplementary Figure 2).

MoBa. For cross sectional analyses, we excluded mothers lacking genetic data, necessary data from the questionnaire completed when study children were aged 14 (self-reported BMI, workplace bullying, or psychological distress), or missing educational qualifications from administrative records. We also excluded a small group (n=344) who responded “not relevant” to workplace bullying question (Supplementary Figure 3).

5. Details of phenotypic variables used in this analysis

Occupation in HUNT. As a socioeconomic indicator in HUNT, we included a 10-group classification of occupational role and sector (categories shown in Table 1), based on the International Standard Classification of Occupations (ISCO-88) (6). This was categorized as: legislators, senior officials and managers; professionals; technicians and associate professionals; clerks; service workers, shop and market sales workers; skilled agricultural and fishery workers; craft and related trade workers; plant and machine operators and assemblers; elementary occupations; armed forces and unspecified occupations. For stratified analyses, this was dichotomized according to skill level as legislators, senior officials and managers; professionals; technicians and associate professionals, and clerks; service workers, shop and market sales workers; skilled agricultural and fishery workers; craft and related trade workers; plant and machine operators and assemblers; elementary occupations. The small group of armed forces and unspecified occupations were excluded from stratified analysis because they could not be classified according to skill broad level https://ilostat.ilo.org/methods/concepts-and-definitions/classification-occupation/#elementor-toc__heading-anchor-6.

Mother’s formal education in MoBa. Mother’s highest level of formal education was derived from administrative records. This was classified according to ISCED categories (primary; lower secondary; upper secondary (basic); upper secondary (final year); post-secondary not higher education; higher education (undergraduate); higher education (graduate level); and higher education (postgraduate)). For analysis, it was treated as a categorical variable with the largest group (undergraduate) as the reference. For stratified analyses, this was dichotomized as: formal education below undergraduate level, and undergraduate level education or higher.

Psychological distress measures. In HUNT, psychological distress was indexed using the 14-item Hospital Anxiety and Depression Scale (HADS)(7). In MoBa, psychological distress was indexed using a scale of 14 selected items from the Hopkins Symptoms Checklist-25 (8), which captures symptoms of depression and anxiety. The 14 items are shown in the documentation for the 14-year Mother’s Questionnaire: https://www.fhi.no/en/ch/studies/moba/for-forskere-

artikler/questionnaires-from-moba/#14-year-olds. In both cases, scores were calculated by summing individual item responses into an overall total. For a small number of participants (<2% of participants in both surveys) with a small amount of missing data (≤2 of 14 items).

6. Details of polygenic scores and two-sample MR analyses

Polygenic scores (PGSs) for BMI were derived using data on single nucleotide polymorphisms (SNPs) which were previously associated with BMI in a genome-wide analysis (GWAS) of 700,000 adults of European ancestry (9). This GWAS identified 941 SNPs independently associated (at $p<1.0\times10^{-8}$) with BMI, of which 934 were available and had passed quality control in HUNT, and 908 in MoBa. PGSs were derived by summing each participant's number of BMI-increasing alleles across these SNPs, multiplied by the corresponding effect size from the GWAS. Details of genotyping and genetic quality control and imputation in HUNT(10) and MoBa(5) are available elsewhere. To account for bias due to ancestral clustering, we included the first 20 principal components of ancestry and genotyping batch as covariates in genetic models. For consistency, these were also included in cross-sectional non-genetic models. For two-sample MR analyses, associations of SNPs in the PGS with WPB were calculated within HUNT and MoBa, and associations of the same SNPs with BMI taken from the GWAS (9).

7. Details of Two-Sample MR Analyses

Using the mrrobust package in Stata, we ran additional MR models using two-sample methodology (Inverse Variance Weighted, MR-Egger, MR-mode and MR-median) which rely on different assumptions and can be used to explore robustness of MR results(11). IVW assumes no horizontal pleiotropy (i.e., that the genetic variants do not influence the outcome other than via the exposure); MR-Egger combines Wald ratio (or ratio estimates) in a meta-regression with an intercept and slope parameter) to estimate the causal effect adjusted for any directional pleiotropy. Here, all SNPs used as instruments can be invalid due to horizontal pleiotropy, so long as the pleiotropy satisfies the Instrument Strength Independent of Direct Effect (InSIDE) assumption. The mode-based estimate requires that the largest number of similar individual instrumental-variable estimates comes from variants which are valid instruments, while MR-median requires that at least 50% of the weight in the analysis stems from variants that are valid. For details of assumptions of all these methods, see https://mr-dictionary.mrcieu.ac.uk.

Supplementary figure 1: Exclusions from the HUNT analytic sample (cross-sectional analyses)

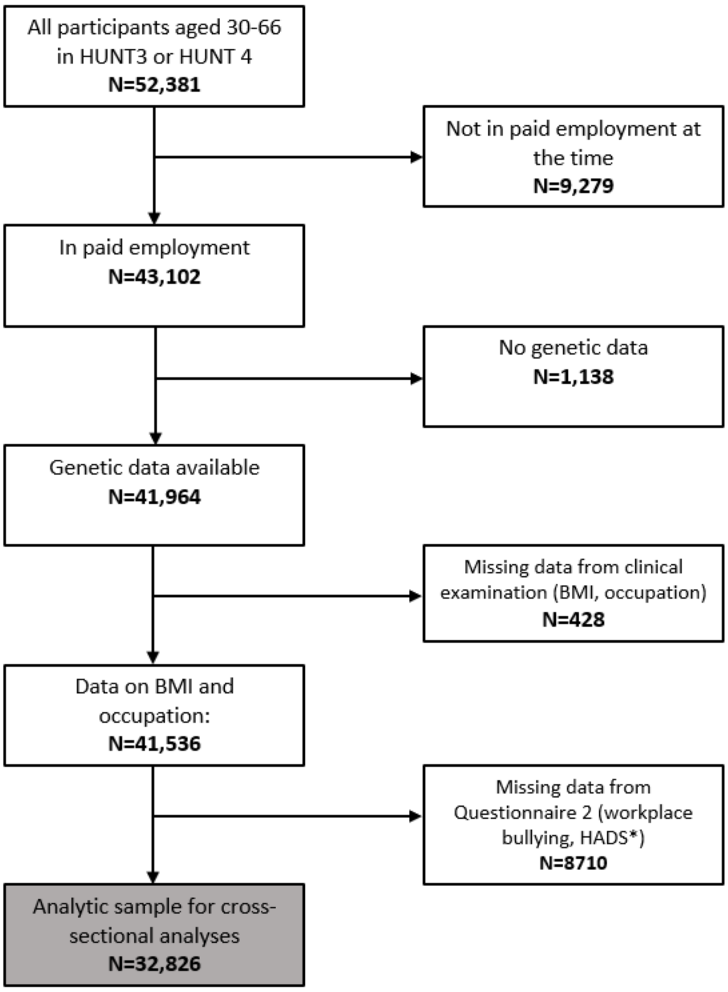

Supplementary figure 2: Exclusions from the HUNT analytic sample (longitudinal analyses)

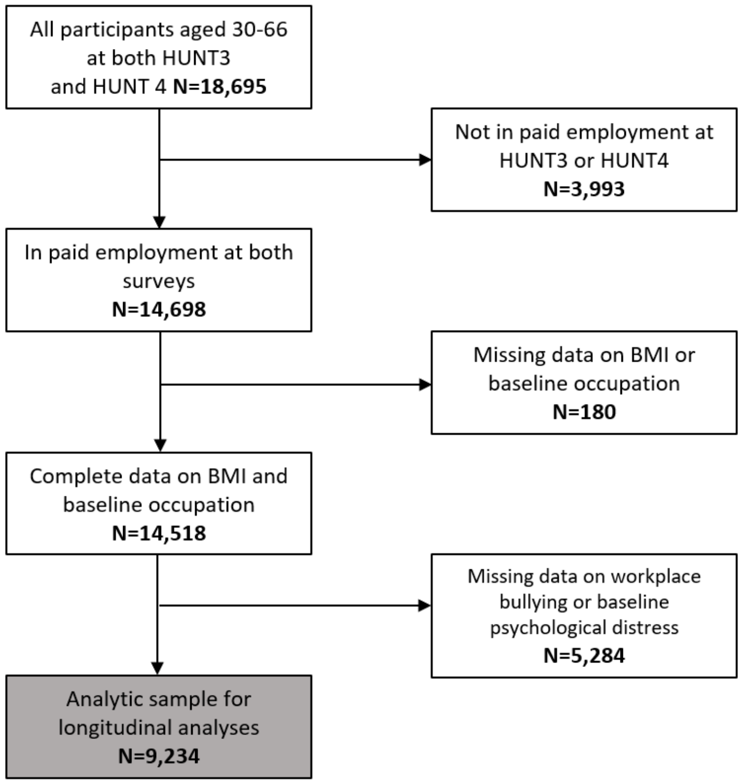

Supplementary figure 3: Exclusions from the MoBa analytic sample (cross-sectional analyses)

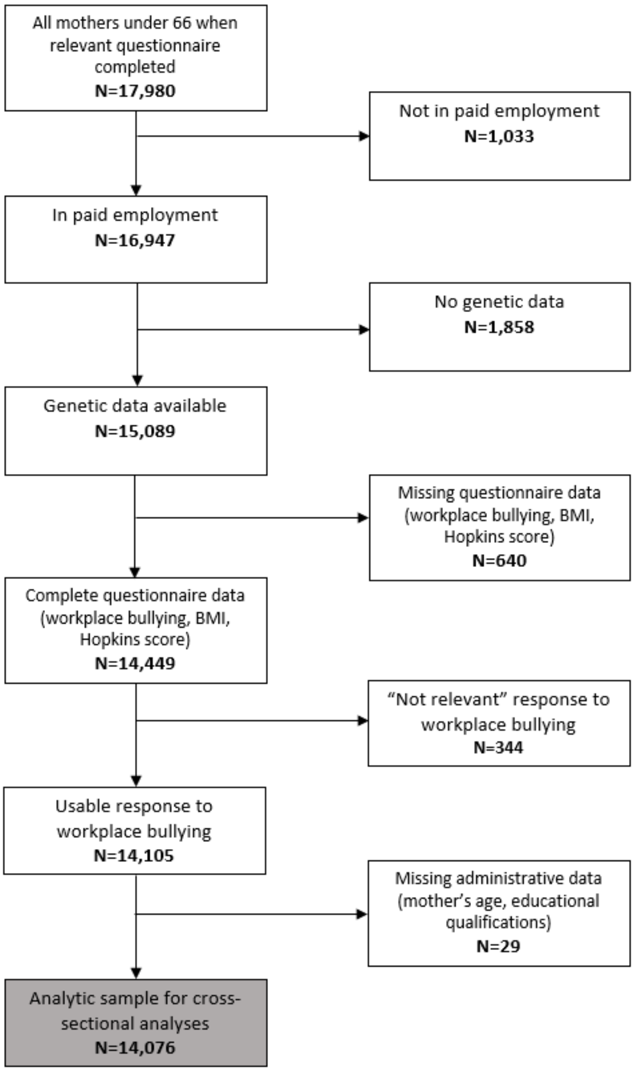

Supplementary Table 1: Descriptive characteristics of HUNT analytic sample for longitudinal analyses (N=9,234)*

	Mean	S.D.	range
Age in years, HUNT3	44.4	6.8	30-57
Age in years, HUNT4	54.9	6.7	40-66
BMI (kg/m^2), HUNT3	26.7	4.1	15.8-54.3
BMI (kg/m^2), HUNT4	27.4	4.4	14.2-51.9
HADS[†] score, HUNT3	6.4	4.9	0-37
			%
Workplace bullying, HUNT3	No, could say never		84.6
	No, rarely		11.9
	Yes, sometimes		3.3
	Yes, often		0.2
Workplace bullying, HUNT4	No, could say never		85.4
	No, rarely		10.3
	Yes, sometimes		4.0
	Yes, often		0.4
Sex	Female		59.8
	Male		40.3
Occupation, HUNT3	Legislators, senior officials and managers		8.4
	Professionals		12.1
	Technicians and associate professionals		26.7
	Clerks		6.7
	Service workers, shop and market sales workers		21.7
	Skilled agricultural and fishery workers		5.8
	Craft and related trade workers		9.0
	Plant and machine operators and assemblers		6.0
	Elementary occupations		3.5
	Armed forces, unspecified occupations		0.2

* All individuals who participated in both HUNT3 and HUNT4, were of working age (<67) and in paid employment at both timepoints, with complete data on exposure, outcome and baseline covariates. [†]Hospital Anxiety and Depression Scale.

**Supplementary Table 2: results of two-sample Mendelian randomization analyses***	
**HUNT (N=32,826)**	Absolute risk difference (%)
Inverse variance weighted (IWV)	0.75 (-0.45, 1.96)
MR-Egger, slope	-1.14 (-2.67, 0.39)
MR-Egger, intercept	0.04 (-0.00, 0.08)
MR-median	0.33 (-1.33, 2.00)
MR-mode	0.05 (-1.62, 1.71)
**MoBa (N=14,076)**	Absolute risk difference (%)
Inverse variance weighted (IWV)	1.13 (-0.32, 2.59)
MR-Egger, slope	0.88 (-0.95, 2.70)
MR-Egger, intercept	0.00 (-0.04, 0.05)
MR-median	1.73 (-0.27, 3.73)
MR-mode	1.08 (-0.93, 3.08)
*Conducted in Stata using mrrobust. Associations of individual SNPs in the PGS with workplace bullying were calculated within HUNT and MoBa, adjusted for age in years, sex (HUNT only), principal components of ancestry, and survey year (HUNT) or year of questionnaire completion (MoBa). Associations of the same individual SNPs with BMI were taken from the GWAS.	

**Supplementary Table 3: cross-sectional associations between BMI and workplace bullying, stratified by possible effect modifiers**					
	Model 1*	Model 2†	Model 2†	Model 2†	Model 3: genetic IV‡
	Absolute risk difference (%)	OR	Absolute risk difference (%)	OR	Absolute risk difference (%)
**HUNT, females (N=18,016)**	0.11 (-0.00,0.22)	1.01 (1.00,1.02)	0.07 (-0.04,0.18)	1.01 (1.00,1.01)	-0.27 (-0. 85,0.31)
**HUNT, males (N=14,810)**	0.59 (0.44,0.75)	1.04 (1.03,1.06)	0.51 (0.36,0.67	1.04 (1.03,1.05)	0.50 (-0.34,1.34)
**HUNT, aged 30-44 (N=11,123)**	0.33 (0.19,0.47)	1.03 (1.01,1.04)	0.21 (0.07,0.35)	1.02 (1.00,1.03)	0.10 (-0.60,0.80)
**HUNT, aged 45-66 (N=21,703)**	0.24 (0.12,0.37)	1.02 (1.01,1.03)	0.23 (0.11,0.34)	1.02 (1.01,1.03)	-0.02 (-0.68,0.63)
**HUNT, occupation: ISCO-88 groups 4, 5, 6, 7, 8, 9§ (N=17,223)**	0.26 (0.13, 0.39)	1.02 (1.01,1.03)	0.24 (0.11,0.36)	1.02 (1.01,1.03)	-0.22 (-0.92, 0.47)
**HUNT, occupation: ISCO-88 groups 1, 2, 3‖ (N=15,428)**	0.22 (0.09, 0.35)	1.02 (1.01,1.03)	0.20 (0.07, 0.32)	1.02 (1.01,1.03)	0.20 (-0.47, 0.87)
**HUNT, 2006-08 survey (N=17,481)**	0.31 (0.17,0.44)	1.02 (1.01,1.03)	0.24 (0.11,0.38)	1.02 (1.01,1.03)	-0.06 (-0.78,0.66)
**HUNT, 2017-19 survey (N=15,345)**	0.24 (0.12,0.37)	1.02 (1.01,1.03)	0.19 (0.07,0.31)	1.01 (1.00,1.02)	0.12 (-0.52,0.75)
**MoBa, aged 30-44 (N=6765)**	0.29 (0.13,0.45)	1.03 (1.01,1.05)	0.22 (0.06,0.38)	1.03 (1.01,1.04)	1.18 (0.36,2.00)
**MoBa, aged 45-60 (N=7311)**	0.24 (0.09,0.40)	1.03 (1.01,1.05)	0.21 (0.05,0.37)	1.02 (1.01,1.04)	0.03 (-0.83,0.89)
**MoBa, formal education below undergraduate level (N=2795)**	0.37 (0.12, 0.62)	1.04 (1.01,1.06)	0.32 (0.07,0.57)	1.03 (1.01,1.06)	1.98 (0.62, 3.33)
**MoBa, formal undergraduate level or higher (N=11,281)**	0.22 (0.10, 0.35)	1.03 (1.01,1.04)	0.18 (0.06, 0.31)	1.02 (1.01,1.04)	0.29 (-0.38, 0.96)
**MoBa, 2016-19 (N=8117)**	0.31 (0.16,0.46)	1.03 (1.02,1.05)	0.27 (0.12,0.42)	1.03 (1.01,1.05)	1.11 (0.25,1.97)
**MoBa, 2020-22 (N=5,899)**	0.23 (0.06,0.40)	1.03 (1.01,1.05)	0.17 (0.00,0.34)	1.02 (1.00,1.04)	0.13 (-0.68,0.93)

*Adjusted for age in years, sex (HUNT only), principal components of ancestry, and survey year (HUNT) or year of questionnaire completion (MoBa). †Model 1 + psychological distress (HADS in HUNT, Hopkins Checklist in MoBa) and socioeconomic indictor (occupation in HUNT, education in MoBa). ‡Adjusted for age in years, sex (HUNT only), principal components of ancestry, genotyping batch, and survey year (HUNT) or year of questionnaire completion (MoBa). §Clerks; Service workers and shop and market sales workers; Skilled agricultural, forestry and fishery workers; Craft and related trades workers; Plant and machine operators, and assemblers; Elementary occupations. ‖Legislators, senior officials and managers; Professionals; Technicians and associate professionals.

**Supplementary Table 4: longitudinal associations between BMI at HUNT3 and workplace bullying at HUNT4, also adjusting for BMI at HUNT2 (N=7,031)**			
**Model 4: Model 3* + BMI at HUNT2**	**N=7,031**	**Absolute risk difference (%)**	**Odds ratio**
Continuous BMI (per kg/m^2)		0.13 (-0.22, 0.48)	1.01 (0.98,1.04)
BMI categories (reference: recommended weight (18.5-24.9kg/m^2))			
Overweight (25.0-29.9kg/m^2)		-0.08 (-2.13, 1.98)	0.99 (0.82,1.20)
Obesity I (30.0-34.9kg/m^2)		3.87 (0.67,7.04)	1.37 (1.03, 1.81)
Obesity II (≥35.0kg/m^2)		-1.16 (-6.75,4.43)	0.87 (0.52,1.48)
Underweight (<18.5kg/m^2		4.46 (-8.00-16.92)	1.37 (0.51, 3.69)
*Adjusted for age in years, sex, principal components of ancestry, genotyping batch, psychological distress (HADS), socioeconomic indictor (occupation), workplace bullying at HUNT3.			

**Supplementary Table 5: longitudinal associations between BMI and workplace bullying in HUNT, stratified by possible effect modifiers**						
Forward: association of BMI in 2006-08 (per kg/m^2) and workplace bullying in 2017-19						
	Model 1*	Model 1*	Model 2†	Model 2†	Model 3‡	Model 3‡
	Absolute risk difference (%)	OR	Absolute risk difference (%)	OR	Absolute risk difference (%)	OR
HUNT, all (N=9,234)						
HUNT, females (N=5517)	0.02 (-0.20,0.23)	1.00 (0.98,1.02)	-0.02 (-0.23,0.20)	1.00 (0.98,1.02)	-0.06 (-0.27,0.15)	0.99 (0.98,1.01)
HUNT, males (N=3717)	0.37 (0.05,0.68)	1.03 (1.00,1.06)	0.32 (0.00,0.63)	1.03 (1.00,1.06)	0.19 (-0.11,0.49)	1.02 (0.99,1.05)
HUNT, aged 30-44 at baseline (N=4676)	0.14 (-0.10,0.37)	1.01 (0.99,1.03)	0.07 (-0.17,0.31)	1.01 (0.99,1.02)	0.02 (-0.21,0.25)	1.00 (0.98,1.02)
HUNT, aged 45-66 at baseline (N=4558)	0.10 (-0.17,0.37)	1.01 (0.99,1.03)	0.09 (-0.17,0.36)	1.01 (0.99,1.03)	0.00 (-0.25,0.26)	1.00 (0.98,1.02)
HUNT, occupation: ISCO-88 groups 4, 5, 6, 7, 8, 9§ (N=4,860)	0.13 (-0.12,0.37)	1.01 (0.99,1.03)	0.11 (-0.14, 0.35)	1.01 (0.99,1.03)	0.03 (-0.21, 0.26)	1.00 (0.98,1.02)
HUNT, occupation: ISCO-88 groups 1, 2, 3‖ (N=4,356)	0.06 (-0.20,0.32)	1.00 (0.98,1.03)	0.04 (-0.22, 0.29)	1.00 (0.98,1.03)	-0.02 (-0.26, 0.23)	1.00 (0.98,1.02)
Reverse: association of workplace bullying in 2006-08 and BMI (per kg/m^2) in 2017-19						
	Model 1*	Model 2†	Model 3:			
	Difference in BMI (kg/m^2)	Difference in BMI (kg/m^2)	Difference in BMI (kg/m^2)			
HUNT, all (N=9,234)						
HUNT, females (N=5517)	0.51 (0.15,0.86)	0.42 (0.06,0.78)	0.20 (0.01.0.40)			
HUNT, males (N=3717)	0.61 (0.28,0.93)	0.50 (0.17,0.84)	0.09 (-0.09,0.26)			
HUNT, aged 30-44 at baseline (N=4676)	0.63 (0.25,1.00)	0.47 (0.09,0.85)	0.19 (-0.05,0.37)			
HUNT, aged 45-66 at baseline (N=4558)	0.49 (0.16,0.82)	0.46 (0.13,0.80)	0.16 (-0.01,0.32)			
HUNT, occupation: ISCO-88 groups 4, 5, 6, 7, 8, 9§ (N=4,860)	0.56 (0.23, 0.89)	0.51 (0.17, 0.84)	0.18 (-0.00, 0.36)			
HUNT, occupation: ISCO-88 groups 1, 2, 3‖ (N=4,356)	0.42 (0.02, 0.78)	0.39 (0.01, 0.78)	0.13 (-0.07, 0.33)			
*Adjusted for age in years, sex (HUNT only), principal components of ancestry, genotyping batch, and survey year (HUNT) or year of questionnaire completion (MoBa). †Model 1 + psychological distress (HADS in HUNT, Hopkins Checklist in MoBa) and socioeconomic indictor (occupation in HUNT, education in MoBa). ‡Model 2 + baseline value of the outcome (workplace bullying, or BMI).						

1. Åsvold BO, Langhammer A, Rehn TA, Kjelvik G, Grøntvedt TV, Sørgjerd EP, et al. Cohort Profile Update: The HUNT Study, Norway. Int J Epidemiol [Internet]. 2023 Feb 1;52(1):e80–91. Available from: https://doi.org/10.1093/ije/dyac095

2. Krokstad S, Langhammer A, Hveem K, Holmen TL, Midthjell K, Stene TR, et al. Cohort Profile: The HUNT Study, Norway. Int J Epidemiol [Internet]. 2013 Aug 1;42(4):968–77. Available from: https://doi.org/10.1093/ije/dys095

3. Magnus P, Birke C, Vejrup K, Haugan A, Alsaker E, Daltveit AK, et al. Cohort Profile Update: The Norwegian Mother and Child Cohort Study (MoBa). Int J Epidemiol [Internet]. 2016 Apr 10;45(2):382–8. Available from: https://doi.org/10.1093/ije/dyw029

4. Paltiel L, Anita H, Skjerden T, Harbak K, Bækken S, Nina Kristin S, et al. The biobank of the Norwegian Mother and Child Cohort Study – present status. Norsk Epidemiologi. 2014 Dec;24(1-2 SE-).

5. Corfield EC, Frei O, Shadrin AA, Rahman Z, Lin A, Athanasiu L, et al. The Norwegian Mother, Father, and Child cohort study (MoBa) genotyping data resource: MoBaPsychGen pipeline v.1. Preprint at https://www.biorxiv.org/content/101101/20220623496289v4 [Internet]. 2024 Jan 1; Available from: http://biorxiv.org/content/early/2022/07/01/2022.06.23.496289.abstract

6. Statistics Norway. Standard for yrkesklassifisering (Standard Classification of Occupations) [Internet]. Oslo–Kongsvinger; 1998. Available from: https://hunt-db.medisin.ntnu.no/hunt-db/pdf/styrk1.pdf

7. Zigmond AS, Snaith RP. The Hospital Anxiety and Depression Scale. Vol. 67, Acta Psychiatrica Scandinavica. United Kingdom: Blackwell Publishing; 1983. p. 361–70.

8. Hesbacher PT, Rickels K, Morris RJ, Newman H, Rosenfeld H. Psychiatric illness in family practice. J Clin Psychiatry. 1980 Jan;41(1):6–10.

9. Yengo L, Sidorenko J, Kemper KE, Zheng Z, Wood AR, Weedon MN, et al. Meta-analysis of genome-wide association studies for height and body mass index in ∼700000 individuals of European ancestry. Hum Mol Genet [Internet]. 2018 Aug 16;27(20):3641–9. Available from: https://doi.org/10.1093/hmg/ddy271

10. Brumpton BM, Graham S, Surakka I, Skogholt AH, Løset M, Fritsche LG, et al. The HUNT study: A population-based cohort for genetic research. Cell Genomics [Internet]. 2022;2(10):100193. Available from: https://www.sciencedirect.com/science/article/pii/S2666979X22001422
